# Versatile Surface Electrodes for Combined Electrophysiology and Two-Photon Imaging of the Mouse Central Nervous System

**DOI:** 10.3389/fncel.2021.720675

**Published:** 2021-08-10

**Authors:** Michael Schweigmann, Laura C. Caudal, Gebhard Stopper, Anja Scheller, Klaus P. Koch, Frank Kirchhoff

**Affiliations:** ^1^Molecular Physiology, Center for Integrative Physiology and Molecular Medicine (CIPMM), University of Saarland, Homburg, Germany; ^2^Department of Electrical Engineering, Trier University of Applied Sciences, Trier, Germany

**Keywords:** liquid crystal polymer electrodes, cortical stimulation, electrocorticogram, *in vivo* two-photon laser-scanning microscopy, neuron-glia interaction, astrocytes

## Abstract

Understanding and modulating CNS function in physiological as well as pathophysiological contexts remains a significant ambition in research and clinical applications. The investigation of the multifaceted CNS cell types including their interactions and contributions to neural function requires a combination of the state-of-the-art *in vivo* electrophysiology and imaging techniques. We developed a novel type of liquid crystal polymer (LCP) surface micro-electrode manufactured in three customized designs with up to 16 channels for recording and stimulation of brain activity. All designs include spare central spaces for simultaneous 2P-imaging. Nanoporous platinum-plated contact sites ensure a low impedance and high current transfer. The epidural implantation of the LCP micro-electrodes could be combined with standard cranial window surgery. The epidurally positioned electrodes did not only display long-term biocompatibility, but we also observed an additional stabilization of the underlying CNS tissue. We demonstrate the electrode’s versatility in combination with *in vivo* 2P-imaging by monitoring anesthesia-awake cycles of transgenic mice with GCaMP3 expression in neurons or astrocytes. Cortical stimulation and simultaneous 2P Ca^2+^ imaging in neurons or astrocytes highlighted the astrocytes’ integrative character in neuronal activity processing. Furthermore, we confirmed that spontaneous astroglial Ca^2+^ signals are dampened under anesthesia, while evoked signals in neurons and astrocytes showed stronger dependency on stimulation intensity rather than on various levels of anesthesia. Finally, we show that the electrodes provide recordings of the electrocorticogram (ECoG) with a high signal-to noise ratio and spatial signal differences which help to decipher brain activity states during experimental procedures. Summarizing, the novel LCP surface micro-electrode is a versatile, convenient, and reliable tool to investigate brain function *in vivo*.

## Introduction

The perpetuate quest of understanding and modulating brain function continuously confronts the scientific community with a multitude of technical challenges (Rusakov, 2015; Chen et al., 2021). Neurons and their electrophysiological characteristics were the long-standing focus of brain research, promoting the development of various electrophysiological probes to record and/ or stimulate brain activity. Applications ranged from single cell experiments, over *ex vivo* slice measurements, to *in vivo* recording and stimulation from the surface or within the tissue. Electrical stimulation of the CNS is used in clinical routines to treat disorders such as Parkinson’s disease by deep brain stimulation or depression by transcranial electrical stimulation (Kirsch and Nichols, 2013; Beudel and Brown, 2016; Beckner, 2020). The interaction of the electrical field and the excitable tissue is the base for artificial stimulation. When artificially triggering action potentials with electrical stimulation, the voltage-dependent conductivity of the cell membrane for Na^+^ (and K^+^) is exploited. The neuronal excitation depends on the applied field and cell orientation, neuronal subtype, cellular structure, as well as the resulting field distribution generated by the anisotropy of the neural tissue (Rattay, 1998; Basser and Roth, 2000; Radman et al., 2009; Ye and Steiger, 2015). However, advances in neuroscience established the essential contribution of glial cells to brain function in health and disease. Glial cells not only crucially maintain brain homeostasis, but actively participate in neurotransmission, shape neural circuits (Parpura et al., 1994; Araque et al., 1999; Verkhratsky and Nedergaard, 2018; Durkee and Araque, 2019; Caudal et al., 2020), act as resident immune-competent cells (Nimmerjahn et al., 2005; Prinz et al., 2019) and provide myelin sheaths, thereby ensuring fast and reliable neuronal communication (Simons and Nave, 2015; Swire and Ffrench-Constant, 2018). Moreover, glial cell (dys-) function has been implicated in a variety of CNS disorders including Alzheimer’s (Gómez-Gonzalo et al., 2017; Arranz and De Strooper, 2019), Parkinson’s (Yun et al., 2018; Guo et al., 2020), multiple sclerosis (International Multiple Sclerosis Genetics Consortium, 2019; Yeung et al., 2019; Traiffort et al., 2020) and epilepsy (Heuser et al., 2018; Nikolic et al., 2018; Deshpande et al., 2020). Importantly, glial cells such as astrocytes, primarily display activation through intracellular Ca^2+^ rises coupled to the release of neuroactive substances, modulating network function (Bazargani and Attwell, 2016; Covelo and Araque, 2018). In contrast, neurons are primarily characterized by their electrical activity but nonetheless exhibit extensive Ca^2+^ signaling relevant in physiology and pathology (Brini et al., 2014). In addition, neuron-glia interactions are governed by second messengers other than Ca^2+^, e.g., Na^+^ (Ross et al., 2013; Karus et al., 2015; Ziemens et al., 2019) and cyclic AMP (Harada et al., 2017; Bernier et al., 2019). Investigation of those second messenger dynamics *in vivo* requires the use of fluorescent indicators and sensors, thus endorsing the refinement and novel development of neuroscientific tools to study neuron-glia interactions by combining electrophysiological and 2P-imaging techniques.

**Figure 1:**
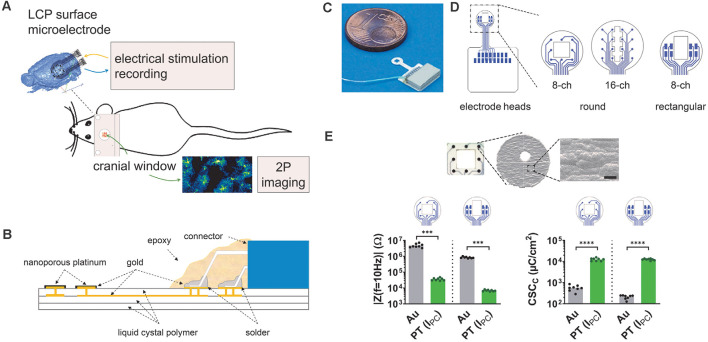
Development of liquid crystal polymer (LCP) surface electrodes for *in vivo* studies combining electrophysiology and imaging. **(A)** Flexible yet robust surface microelectrodes, applicable for *in vivo* investigation of electrical brain stimulation, electrocorticographic recording of brain activity, and 2P-imaging of Ca^2+^ signals. **(B)** Electrode structure with an LCP base including golden electrical structure and superficial gold electrodes, electroplated with nanoporous platinum. The nano-connector is soldered to the terminal pads and secured with epoxy resin. **(C)** Fully assembled LCP surface microelectrode. **(D)** Three different electrode arrays with eight or 16 circular electrodes (3 mm or 4 mm in diameter) for ECoG recording or eight rectangular electrodes for stimulation. Circular electrode sites are 150 μm in diameter; the size of rectangular-shaped sites is 400 μm × 200 μm. The two 8-channel electrode configurations have a spare square in the center (1 mm × 1 mm or 1 mm × 1.5 mm), the 16-channel electrode has a polygon (length 2.8 mm; small width: 400 μm; large width: 800 μm), allowing 2P-imaging. **(E)** Top: scanning electron micrograph of a single electrode site showing the surface structure, scale bar = 5 μm. Bottom: typical magnitude of electrode impedance at a frequency of 10 Hz (|Z(*f* = 10 Hz)|) and typical cathodic charge storage capacity (CSC_C_). Compared with the gold electrode sites, platinum-coated sites display a lower impedance enabling recordings with less noise and a higher CSC_C_ allowing for higher stimulation currents. Statistics: | Z(*f* = 10 Hz) |: Mann–Whitney test and CSC_C_: unpaired *t*-test ***p < 0.001, ****p < 0.0001. *N* = 8 measurements per condition.

Considering combined electrophysiology and 2P-imaging *in vivo* (Figure 1A), the requirements for microelectrodes are ample (Chen et al., 2021). First and foremost, the electrode material must be biocompatible for chronic use, without mechanically damaging the tissue or eliciting inflammatory responses from glial cells. Along the same lines, the electrode array application should be minimally invasive and preferentially keep the dura mater intact (surface electrodes) without entailing major compromises on spatial resolution. Next, the electrode array should be either highly transparent (Park et al., 2014) or provide an optical window to perform imaging (multiphoton, coherent anti-stokes Raman spectroscopic microscopy etc.), potentially coupled to optogenetic approaches (Park et al., 2014; Xie et al., 2020). However, the biomaterial should be stable yet flexible for optimal tissue contact and withstand the impact of laser radiation during 2P-imaging without excessive heat generation. From an electrochemical point of view, the electrodes should be optimized, providing low impedance and high current transfer capability (Cogan, 2008). Finally, the fully assembled microelectrode should allow a safe, fast, and reproducible application in common (rodent) model organisms and come at an affordable cost.

Polyimide (PI) and LCP are the most common flexible carrier materials of industrial large-scale production. Their electrical properties in combination with their chemical and mechanical strength make both materials good candidates for microelectrode assemblies, with LCP electrodes being significantly less expensive than PI electrodes (Woods et al., 2018). Here, we developed LCP electrodes of a stacked support structure formed by Ultralam 3850 sheets and Ultralam 3908 bonding film (Rogers Corporation; Woods et al., 2018). Such LCP material has also been used to record acute neuronal activity in humans (Chiang et al., 2021), indicating its high biocompatibility.

## Materials and Methods

### Electrode Technology

The electrodes were designed based on triple LCP layers (Dyconex AG, Switzerland) allowing flexibility, yet maintaining stability, while the electrical circuit structure was made of a double gold layer (Figure 1B). The LCP structure had a thickness of about 75 μm, a limitation in the minimum electrode site size of about 100 μm, and an interconnection width of about 30 μm. The PCB layout program EAGLE (version 7.5.0 Light; CadSoft) was used to generate the electrode geometry and electrical layout, which was sent to the manufacturer for large-volume industrial production. Epoxy resin (TC-EP05-24, TOOLCRAFT) was used to cover the connector (NPD-18-18AA-GS, Omnetics Connector Corp.) and pads. The gold electrode sites were coated with galvanized nanoporous platinum (5 g hexachloroplatinic acid dissolved in 375 ml distilled water; pulse electroplating at 0.3 kA/m^2^, 90 pulses) and electrochemically characterized by determining the electrode impedance and the cathodic charge storage capacity (CSC_C_; Cogan, 2008) with the multi-chemistry device Gamry Interface 1000 (Gamry Instruments).

### Epidural Electrode Implantation and Cranial Window Surgery

Animals were anesthetized with a mixture of 2% Isoflurane with O_2_ (0.6 L/min) and N_2_O (0.4 L/min) and kept on a heating plate. A standard craniotomy (3–4 mm in diameter; Cupido et al., 2014; Kislin et al., 2014) was performed over the somatosensory cortex and the surface electrode was placed on the dura mater before applying the glass coverslip. A ground electrode (platinum wire) was superficially inserted into the cerebellar vermis and fixed to the skull surface with dental cement. Electrodes, glass coverslips, and platinum wire were disinfected and cleaned with alcohol (70% ethanol) and distilled water. Finally, a 3D-printed custom-made holder for head restraining was applied and all components were fixed with dental cement.

### *In vivo* Two-Photon Laser Scanning Microscopy (2P-LSM)

Images were acquired on a custom-made 2P-LSM setup with a mode-locked Ti:Sapphire laser (Vision II, Coherent) using ScanImage software (Pologruto et al., 2003). 2P-LSM settings: laser power 30–50 mW; frame rate of 1.9 Hz [field of view (FOV) size 256 μm × 256 μm] or 3.3 Hz (FOV size 256 μm × 256 μm); pixel size: 0.5 μm × 0.5 μm; astroglial Ca^2+^-transients were acquired in cortical layer I (40 μm–90 μm) and neuronal activity in layer II/III (180 μm–210 μm. Prior to Ca^2+^ imaging, animals were habituated according to adapted protocols without water restriction (Guo et al., 2014; Kislin et al., 2014). During imaging, the animals were head-fixed and anesthesia was delivered *via* a breathing mask [0–2.5% isoflurane with O_2_ (0.6 L/min) and N_2_O (0.4 L/min)].

### Automated Detection of Spontaneous Ca^2+^ Events

Ca^2+^-event analysis was performed using a custom-made analysis software based on MATLAB (MSparkles, unpublished). First, the fluorescence range of each pixel along the temporal axes of the image stack was computed. Next, local maxima within the range projection were used as seed points for simultaneous, correlation-based region growing. Thereby, the temporal correlation of a candidate pixel with the corresponding seed point was computed using Pearson’s linear correlation coefficient. A user-definable correlation threshold was set as the stopping criterion of the region growing process in case the temporal evolution of a candidate pixel deviated too strongly from its respective seed point. A pixel-based correlation coefficient maximization algorithm was employed for image registration (Evangelidis and Psarakis, 2008) and to reduce small motion artifacts of the acquired time series. Upon ROI integration (computing the mean fluorescence per ROI per image), properties of Ca^2+^ transients were obtained using the MATLAB function *findpeaks()*.

### Electrocorticographical Recording

A 16-channel biosignal amplifier (g.USBamp, g.Tec medical engineering) with a preamplifier (g.HEADstage, gTec medical engineering) and a custom made control software based on MATLAB/Simulink (Englert et al., 2017) was used to acquire the electrical biosignals at a channel sampling rate of 1.2 kHz. Filtering was chosen with a band pass filter of 0.5 Hz to 250 Hz and a notch filter of 50 Hz. Signal processing was performed by calculating the short-time Fourier transform (rectangular sliding window of 2 s shifted in steps of 1 s) and by calculating the correlation coefficient between all single recording channels with customized MATLAB scripts using the main functions *spectrogram() and corr(), respectively*. ECoG sections of 30 s from awake and anesthetized mice were used to calculate channel correlation and analyzed as a function of electrode distances. In addition, the channel correlation was calculated with a rectangular sliding window of 2 s in steps of 1 s.

### Electrical Stimulation

A single channel stimulator (ISO-STIM 01D, NPI electronic) connected *via* a digital-to-analog output card (NI PCI-6723, National Instruments) was controlled by a customized control software (LabView, National Instruments). A second output channel of the analog output card was used for synchronization with the 2P-microscope. The output sampling rate was set to 100 kHz. Threshold (Th) definition was performed in anesthetized animals (isoflurane concentration 1.5%) at 50 Hz stimulation frequency by increasing stimulation current intensities in steps of 25 μA (starting at 100 μA) for 300 frames (approximately 90 s). Threshold values typically ranged from 125–200°μA. Subsequently, we applied two stimulations at Th, Th + 50 μA, Th +100 μA to quantify the Ca^2+^-responses. For signal analysis, the mean value per statistical parameter of both stimulation per current strength was calculated. The stimulation series was applied to anesthetized (isoflurane concentrations: 1.5%, 1%, 0.5%) and awake (isoflurane concentrations: 0%) mice.

### Detection of Electrically Evoked Ca^2+^ Events

Custom-made MATLAB scripts were used to analyze the Ca^2+^ events in neurons and astrocytes. A pixel-based correlation algorithm to align individual images was employed for stack registration (Evangelidis and Psarakis, 2008) followed by the calculation of the mean fluorescence intensity of the whole individual gray-scale images [MATLAB function *mean()*]. Peak amplitude (*F* – *F*_0_/*F*_0_), delay to peak, given as time from 10% of peak amplitude (rising signal) to peak amplitude, and signal duration, given as time from 10% to 10% of peak amplitude, were determined as parameters of the transient signals. Only Ca^2+^ events that followed from electrical stimulation were considered.

### Immunohistochemistry

Animals were perfused intracardially with PBS and 4% formaldehyde. Dissected brains were subsequently post-fixed overnight at 4°C. Free-floating coronal vibratome slices (40 μm) were generated (VT1000S, Leica, Biosystems, Wetzlar, Germany) and sections were collected, blocked, and permeabilized (blocking solution, 0.5% Triton X-100 and 5% horse serum in PBS) for 1 h at RT. The slices were incubated with primary antibodies diluted in blocking solution overnight, at 4°C. After washing with PBS, the slices were incubated with fluorescent secondary antibodies diluted in blocking solution for 2 h at RT. DAPI (0.025 μg/ml final concentration) was added to the secondary antibody solutions to stain nuclei. The primary antibodies were used as follows: goat anti-GFP (1:1,000, Rockland, Limerick, PA, USA), mouse anti-GFAP (1:500, Novocastra, Leica Biosystems, Wetzlar, Germany) and rabbit anti-Iba1 (1:500, Wako, Osaka, Japan). Stained slices were scanned with the fully automated epifluorescence slide scanner microscope AxioScan.Z1 (Zeiss, Oberkochen, Germany) and analyzed with the ZEN imaging software (Zeiss). Cortical areas (3.92 ± 0.32 mm^2^) on both hemispheres of the slices were selected for analysis of the fluorescence intensities. The same area size was used for all stainings of one slice.

### Statistics

Statistical analysis was performed with GraphPad Prism 8. Data distributions were assessed with the Shapiro-Wilk normality test. In the case of normal distribution, single comparisons were computed with parametric unpaired *t*-tests and multiple comparisons with two-way ANOVA (mixed model) followed by Tukey’s* post hoc* test. When data were not normally distributed, we applied a non-parametric Mann–Whitney test for single comparisons and Kruskal–Wallis test followed by Dunn’s* post hoc* test for multiple comparisons.

### Animals

Mice were maintained in the animal facilities of the Centre for Integrative Physiology and Molecular Medicine (CIPMM, University of Saarland). Mice received food *ad libitum*. Knockin GLAST-Cre^ERT2^ mice (Slc1a3^tm1(cre/ERT2)Mgoe^, MGI:3830051; Mori et al., 2006) and knockin Nex-Cre mice (Neurod6^tm1(cre)Kan^, MGI: 2668659) were crossbred to mice with Rosa26 reporter mice (Gt(ROSA)26Sor^tm1(CAG-GCaMP3)Dbe^, MGI: 5659933 (Paukert et al., 2014). For *in vivo* 2P-LSM, ECoG recording, stimulation experiments, and IHC, 12–17 week old mice were studied. To induce reporter expression in 8 week old transgenic GLAST-Cre^ERT2^ mice, animals received tamoxifen intraperitoneally (10 μg/ml, 100 μl/10 g body weight) once per day for five consecutive days (Jahn et al., 2018).

### Ethics Statement

Animal experiments were carried out at the University of Saarland according to European and German guidelines and approved by “Landesamt für Gesundheit und Verbraucherschutz” of Saarland state (license numbers: 71/2013, 36/2016).

## Results

### Surface Electrode Arrays for Electrical Stimulation and ECoG Recording Enclosing an Optical Window

Our aim was to develop microelectrodes, applicable for *in vivo* measurements of electrical brain stimulation as well as the electrocorticographic recording of brain activity coupled to 2P-imaging in the region of interest (Figure 1A). Thereby, minimizing electrode effects on the tissue is pivotal for the functional assessment of complex network interactions. To avoid cellular responses associated with penetrating electrodes (Burda et al., 2016; Sohal et al., 2016; Donat et al., 2017), three different LCP surface electrodes were developed to be placed on the dura, enabling stimulation and recording of the brains’ electrical activity (Figure 1A). The electrode base consists of a triple layer of white LCP, accommodating the two-layered golden electrical structure. The inner gold layer served as an interconnection plane, the outer layer harbors the electrode sites and solder pads. Electrode sites were electroplated with nanoporous platinum. An 18-pin nano-connector was soldered to the terminal pads and fixed by epoxy resin (Figure 1B).

Each electrode can potentially be used for stimulation and recording. Here, we used preferentially an eight-channel model with circular electrode sites (diameter = 150 μm) arranged in a three-by-three matrix (center-center distance: 750 μm) with sparing the center, for recordings (Figures 1C,D, rec) and an eight-channel model with rectangular electrodes sites (400 μm × 200 μm) arranged in two groups of four for stimulation (Figure 1D, stim). The round electrode diameter was 3 mm and both electrodes contained a central window for 2P-LSM (size: 1 mm × 1 mm or 1 mm × 1.5 mm respectively; Figures 1C,D). In addition, a 16-channel electrode with round-shaped sites (diameter = 150 μm; center-center distance = 750 μm; arranged in a four-by-four matrix) was developed for the acquisition of electrocorticograms (ECoGs) over a larger cortical region. The optical window of the electrode has a polygonal shape (length 2.8 mm; small width: 400 μm; large width: 800 μm; Figure 1D). The distance between electrode sites and optical window, as well as the sizes of round electrode sites were chosen in accordance with the manufacturer’s minimum recommendations. The catwalk of each electrode array had a length of 3 mm and a width of 1.1 mm and allowed the placement of the electrode within the craniotomy. The pad area accommodating the connector was 8.2 mm × 7.4 mm. Prior to covering the connector with epoxy resin, an additional wire was soldered to the electrode connector enabling the use of an additional ground electrode (Figure 1C).

After platinum electroplating of the electrode sites (Figure 1E), the impedance magnitude at a frequency of 10 Hz was reduced from 3.3 MΩ–7.2 MΩ to 29 kΩ–48 kΩ for the round-shaped sites and 740 kΩ–1 MΩ to 6 kΩ–8.2 kΩ for the rectangular-shaped sites. This resulted in lower noise (Obien et al., 2014) and a lower voltage drop during stimulation. The cathodic charge storage capacities, as a comparable measure of stimulation current drive capability, were increased from 420 μC/m^2^ to 550 μC/m^2^ to 10,500 μC/cm^2^ to 15,800 μC/cm^2^ (round electrode sites) and from 130 μC/m^2^ to 250 μC/m^2^ to 10,500 μC/m^2^ to 14,000 μC/m^2^ (Figure 1E). The rectangular electrode sites can either be used in single electrode configuration for a higher stimulation selectivity or connected together for generating a broader and more uniform current distribution.

### LCP Surface Electrodes Are Highly Biocompatible and Even Stabilize the Tissue After Cranial Window Surgery

**Figure 2:**
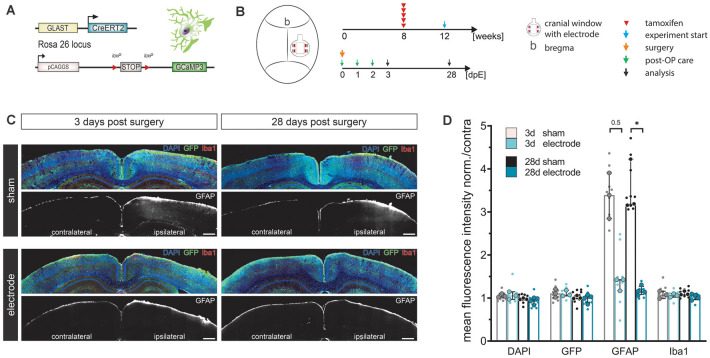
LCP surface electrodes are long-term biocompatible and favorable for *in vivo* studies. **(A)** The biocompatibility study was carried out on transgenic mice with tamoxifen-induced, astrocyte-specific GCaMP3 expression. **(B)** Eight-week-old animals were administered five consecutive i.p. injections of tamoxifen to induce GCaMP3 expression in astrocytes. At 12 weeks of age, animals underwent cranial window surgery with or without (sham) surface electrode implantation. An immunohistochemical analysis was carried out 3 or 28 days post-surgery. **(C)** Activation of microglia (Iba1) and astrocytes (GFAP) was assessed by immunohistochemical staining of coronal slices. GCaMP3 expression was revealed by GFP immunostaining. Ipsilateral: surgery side, contralateral: control side. Scale bars indicate 500 μm. **(D)** Astroglial GFAP reactivity was increased in sham groups compared to electrode implanted groups both 3 and 28 days post-surgery, while the astrocyte activation of the electrode implanted groups was only minimally increased, indicating high biocompatibility and an additional stabilizing effect of the LCP cortical surface electrode. Ipsilateral fluorescence intensity values were normalized to the contralateral side. Statistics: Kruskal–Wallis test followed by Dunn’s multiples comparisons test *p < 0.05. Data are displayed as median with IQR. Larger data points correspond to the average of slices from the same animal, smaller data points indicate individual slices. 3 d sham *N* (animal) = 3, *n* (slice) = 9; 3 d electrode *N* = 3, *n* = 9; 28 d sham *N* = 3, *n* = 9; 28 d electrode *N* = 4, *n* = 12.

Reactive astrocytes and microglia are primary and reliable indicators of CNS inflammation at different time scales. While microglia respond rapidly, within minutes to hours, astrocytes are activated after a couple of days. After an acute insult of sufficient magnitude or continuous inflammatory processes, both cell types contribute to the development of gliosis (glial scar; Burda and Sofroniew, 2014). Considering that chronic *in vivo* experiments usually start after several days of recovery, we investigated the biocompatibility of the LCP surface electrodes in transgenic mice with astroglial GCaMP3 expression (Figure 2A) 3 days (for acute responses) and 28 days (for chronic responses) post-implantation (Figure 2B). LCP surface electrodes were implanted after a standard craniotomy, covered with a glass coverslip, and histological outcomes were compared to sham-treated mice that only underwent cranial window surgery (Figure 2B).

Cortical cell responses were assessed by fluorescence intensity (FI) analysis of immunohistochemical markers for microglia (Iba1) and reactive astrocytes (GFAP; Figure 2C). The density of recombined astrocytes was assessed by immunodetection of GCaMP3 using GFP antibodies. The general cell density was visualized by nuclear DAPI staining. No alterations of cortical layer structures were detected in either group at any time point (Figure 2C). Ipsilateral FI values normalized to the contralateral side showed that neither the surgery nor the electrode itself changed expression levels of DAPI, GFP, or Iba1 at three or 28 days post-surgery (FI normalized to contra ≈ 1, Figures 2C,D). In contrast, GFAP levels in sham operated animals tripled compared to the contralateral side as well as compared to electrode-implanted animals at both three (FI normalized to contra = 3.4) and 28 days post-surgery (FI normalized to contra = 3.2, *p* = 0.031; Figures 2C,D). Animals carrying the LCP surface electrode displayed a slight GFAP increase 3 days post-surgery (FI normalized to contra = 1.4) that was negligible 28 days post-surgery (FI normalized to contra = 1.2; Figure 2D). We concluded that the LCP surface electrode did not elicit significant activation of microglia or astrocytes at an acute or chronic time scale. In fact, the electrode had an alleviating effect on cortical glia activation compared to the simple cranial window surgery, suggesting an additional stabilizing effect.

### Electrical Stimulation Synchronized With *In vivo* 2P-Ca^2+^ Imaging Reveals Stimulation Intensity and Anesthesia-Dependent Biphasic Neuronal Ca^2+^-Signal Signature

**Figure 3:**
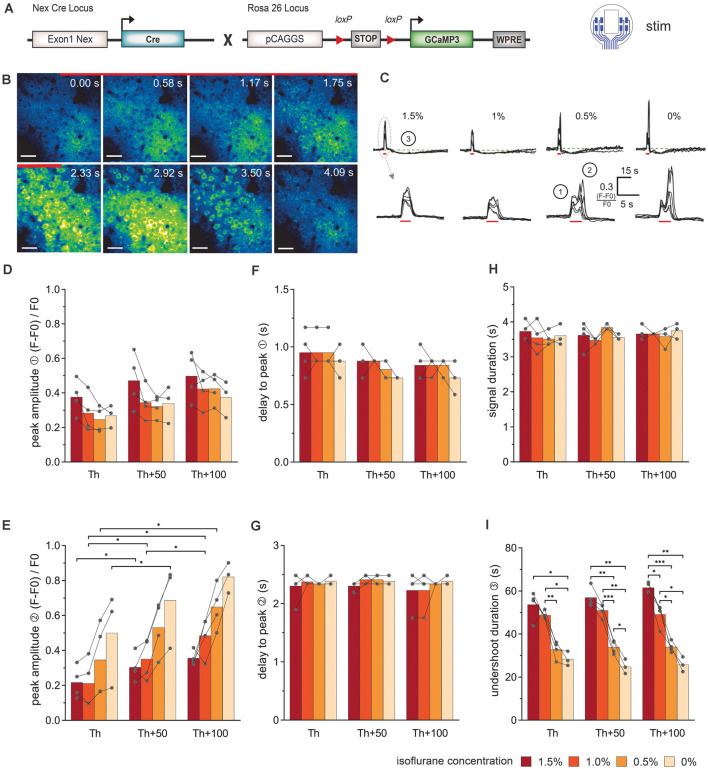
Simultaneous electrical stimulation and *in vivo* 2P-Ca^2+^ imaging reveals the stimulation intensity- and anesthesia-dependent neuronal Ca^2+^-signal signature. **(A)** Transgenic mice expressing the Ca^2+^ sensor GCaMP3 in cortical neurons were used for stimulations with the 8-channel stimulation electrode. **(B)** 2P-Ca^2+^ imaging sequence during electrical stimulation (red bar). The first Ca^2+^ response originates from the neuropil (0–1.17 s) and was followed by transient somatic signals (1.75–3.5 s), scale bar = 40 μm. **(C)** Top row: neuronal Ca^2+^ peaks in response to electric stimulation (red bar) are systematically followed by an undershoot (3) at all isoflurane concentrations (1.5–0%), scale bar = 15 s. Bottom row: at higher temporal magnification, a biphasic peak (1, 2) was reliably observed at isoflurane concentrations ≤0.5%. **(D)** The amplitude of the first neuronal Ca^2+^ peak component was independent of stimulation intensity and anesthesia level. **(E)** The amplitude of the second peak component was potentiated with increasing stimulation intensities. **(F)** The delay between the stimulation onset and the local maximum of the first peak component did not change with stimulation intensity or anesthesia level. **(G)** The delay between the stimulation and the local maximum of the second peak component remained unchanged with varying stimulation intensity or anesthesia level. **(H)** Ca^2+^ peak duration did not vary with stimulation intensities and isoflurane concentrations. **(I)** Duration of the undershoot following the biphasic peak was strongly dependent on the level of anesthesia, with approximately 50% shorter durations in awake animals compared to 1.5% isoflurane anesthesia, across stimulation protocols. Statistics: Two-Way ANOVA (mixed model) followed by Tukey’s multiple comparisons test *p < 0.05, **p < 0.01, ***p < 0.001, for isoflurane 1.5–0.5% *N* = 4 and isoflurane 0% *N* = 3 measurements per stimulation intensity. Th: threshold.

Electrical stimulations were performed in Nex-Cre × GCaMP3 mice (Figure 3A) at different isoflurane concentrations to investigate how anesthesia could affect the Ca^2+^ response of neuronal networks. The surface electrode with rectangular-shaped sites was used (Figure 3A, stim). The influence of the imaging position with reference to the electrode sites was minimized by simultaneously using the two inner electrode sites left and right of the observation window to apply an electrical current. Layer II/III of the cortex were recorded by 2P-LSM at a depth of 180 μm–210 μm. Threshold (Th) definition was performed in anesthetized animals at 50 Hz stimulation until a large Ca^2+^ wave was induced. Subsequently, two 50 Hz stimulations at Th, Th + 50 μA, Th +100 μA were applied to study the elicited Ca^2+^ responses.

The stereotypical neuronal Ca^2+^ waves could be elicited across all levels of anesthesia (Figures 3B,C). Under lower anesthesia (≤0.5%), two peaks became apparent in the Ca^2+^ transients, after extracting the image brightness (Figure 3C). The sources of these peaks were estimated from the short image series of every second 2P-LSM image (Figure 3B). The image at time 0 s was set as the image directly at stimulation onset. Two frames later (1.17 s), the activation of the neuropil was clearly visible (first peak in the Ca^2+^ transients in Figure 3C①), before the somata were activated (second peak, Figure 3C②). The response of the neuropil did not show any dependence on the anesthesia level or stimulation amplitude (Figure 3D). In contrast, the somatic Ca^2+^ transient amplitude varied with isoflurane and strongly depended on the stimulation intensity (Figure 3E). The delay times (mean ± SD) from stimulation onset to maximum image intensities (peak) were rather constant for all measurements (delay peak I: 0.86 s ± 0.13 s; delay peak II: 2.34 s ± 0.16 s; Figures 3F,G). The duration of the Ca^2+^ signal including neuropil and somata was 3.63 s ± 0.24 s and did not respond to the variations of isoflurane or stimulation amplitude (Figure 3H). After each stimulation, an undershoot of fluorescence intensity occurred (Figure 3C③). The undershoot duration decreased from 57.4 s ± 5.5 s to 33.5 s ± 3.0 s with decreasing anesthesia level, but was independent of the stimulation strength (Figure 3I).

### Evoked Astroglial Ca^2+^ Signals Display Stimulation Intensity-Dependent Characteristics

**Figure 4:**
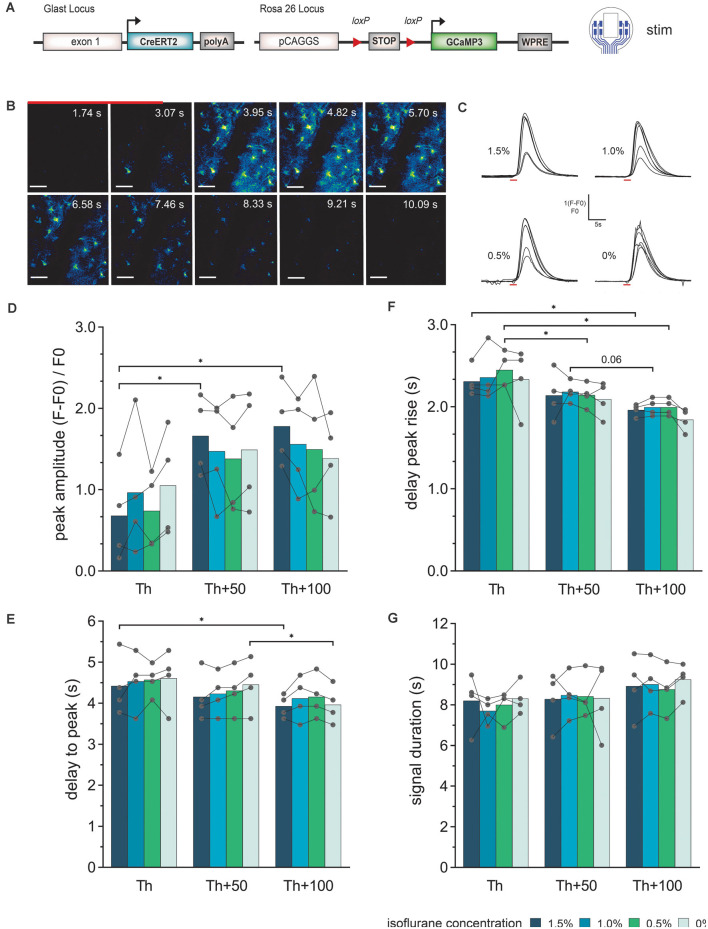
Astroglial Ca^2+^-signal elicited by electrical stimulation *via* the LCP surface electrodes and recorded by *in vivo* 2P imaging. **(A)** The experiment was performed in transgenic mice expressing the Ca^2+^ sensor GCaMP3 in astrocytes and cortical stimulations were applied with the 8-channel stimulation electrode. **(B)** 2P-Ca^2+^ imaging sequence during electrical stimulation (red bar), scale bar = 50 μm. **(C)** Astroglial Ca^2+^ transients in response to electric stimulation (red bar) display a single, uniform peak at all tested isoflurane concentrations (1.5–0%). **(D)** The Ca^2+^ peak amplitude increases with increasing stimulation intensity, especially at high isoflurane concentrations (1.5%), while no significant effect of changing anesthesia levels at the same stimulation intensity could be detected. **(E)** The delay from stimulation onset to Ca^2+^ peak decreases with increasing stimulation intensities but is independent of isoflurane levels. **(F)** Rise of the Ca^2+^ transient to 10% of its maximum was reduced by increasing the stimulation current without changing at varying anesthesia levels of the same current intensity. **(G)** The astroglial Ca^2+^ signal duration was independent of stimulation intensity and anesthesia. Statistics: Two-Way ANOVA followed by Tukey’s multiple comparisons test *p < 0.05, *N* = 4 measurements per stimulation intensity. Th, threshold.

Electrical stimulations were also performed in GLAST-Cre^ERT2^ × GCaMP3 mice to study the response of astrocytes (Figure 4A). The stimulation procedure was chosen as described above (different levels of isoflurane concentration: 1.5%, 1.0%, 0.5%, and 0%; electrode with rectangular-shaped sites (Figure 4A, stim), the inner electrode sites were electrically connected; 50 Hz stimulation; 100 pulses). 2P-LSM recordings were performed in layer I of the cortex at a depth of 40 μm–90 μm. Threshold (Th) definition was performed in anesthetized animals at 50 Hz stimulation until a Ca^2+^ wave was visible. Subsequently, we applied 50 Hz stimulations at Th, Th + 50 μA, Th +100 μA to study the elicited Ca^2+^ responses.

Ca^2+^ transients in cortical astrocytes (somata and processes) could be provoked by electrical stimulation at all levels of anesthesia (Figures 4B,C). In general, astrocytes responded stronger to the stimulation amplitude rather than isoflurane concentration. The astroglial peak amplitudes (Figure 4D) showed an increase from Th to Th + 50 μA, while the Ca^2+^ signal amplitude was rather constant from Th + 50 μA to Th + 100 μA. However, the amplitude values showed high variability. The peak of the astrocytic Ca^2+^ related signals was detected after the end of the stimulation (Figure 4E). There was a slight reduction in the delay time from stimulation onset until the maximum was reached with increasing stimulation intensity. For the highest stimulation current, the delay time was approximately within 3.5 s to 5 s and for the lowest stimulation current approximately 3.5 s to 5.5 s. The delay time from stimulation onset to the Ca^2+^ transient rise (10% of maximum) was decreased by the duration of 1 to 2 imaging frames (300 ms to 600 ms) with increasing stimulation strength (Figure 4F). No significant difference in the duration of the Ca^2+^ transient was visible, neither with the stimulation amplitude nor with the depth of anesthesia (Figure 4G).

### Recordings With the LCP Electrode Arrays Showed a High Signal-to-Noise Ratio and Spatial Signal Differences Reflecting the Brains’ Activity States

**Figure 5:**
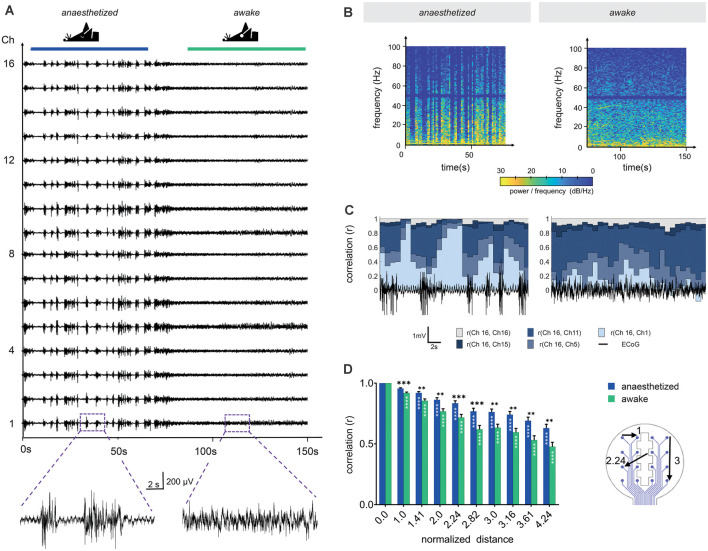
Channel correlation analysis detects differences in brain activity between the anesthetized and the awake state. **(A)** Sixteen-channel ECoG recording displays the pattern change from the anesthetized state (1.5% isoflurane) to the awake state. **(B)** Spectrograms in anesthetized and awake conditions represent the power spectral densities of individual frequencies over time. In the anesthetized animal, frequencies below 10 Hz display the highest power with intermittent synchronous activity bursts, raising the power of frequencies up to approximately 100 Hz. Similarly, frequencies below 10 Hz dominated in the awake state, however, higher frequencies were continuously represented. We applied a Notch filter at 50 Hz, represented by the dark band in this frequency range. **(C)** Channel similarity revealed by correlation analysis between the different electrode sites (channels) and under anesthetized and awake conditions. The similarity of the most distant recording sites [r (Ch16, Ch1)] increased substantially after synchronous burst activity under anesthesia. **(D)** Channel similarity (correlation) decreases with increasing distance between electrode sites (channels). In awake animals, the channel correlation is systematically lower than under anesthesia. Statistics: Two-Way ANOVA followed by Dunnett’s multiple comparisons test for different distances compared to distance 0.0 of the respective anesthesia group (white stars inside bars) and Fisher’s LSD for comparisons between awake and anesthetized (black stars above). Data are shown as mean ± SEM, **p < 0.01, ***p < 0.001, ****p < 0.0001, *N* (anesthetized) = 21 and *N* (awake) = 23 measurements per distance value.

The 16-channel electrode was used to evaluate the recording quality and to assess the spatial distribution of signal events. The depth of anesthesia was modulated (isoflurane 1.5% to 0%) to vary the pattern of the bioelectrical activity (Land et al., 2012). The recordings showed a typical synchronous burst activation of neurons when the mice were anesthetized (Figure 5A). With decreasing anesthesia, the time between the bursts became shorter and the signal amplitude lower, indicating a loss of synchrony. When the mouse was awake, the burst pattern was no longer visible. The difference in neuronal activity was also visible in the spectrograms of the signals (Figure 5B). In the anesthetized mouse, the highest signal intensities were at frequencies below 10 Hz, and frequencies higher than 30 Hz were temporarily visible when the neurons fired synchronously. Since there was no evidence of signal components with a broad frequency range during the burst suppression phases, we concluded that there was no detectable noise affecting the signal quality. In the awake state, the highest signal intensity was also below 10 Hz, but the higher frequencies were always present due to the continuous spiking activity (Figure 5B).

The individual time of signal traces showed a high similarity between the individual channels (Figure 5A). To characterize the channel similarity, the correlation between the recorded signals was calculated. A stepwise calculation (sliding window of 2 s, step time 0.5 s) demonstrated the dependency on the synchrony of cortical electrical activity (Figure 5C). The channel similarity was lower if there was high spike activity (bursts and neuronal activity in the awake state). In addition, the correlation decreased with channel distance. Correlations were calculated for 30 s traces in relation to the distance of the electrode sites (Figure 5D). The distances of the electrode sites are given as normalized geometric distances (value of 1 means the electrode center-center distance of 750 μm, 1.41 means 1057.5 μm, etc.). This diagram confirms the results from the stepwise calculation. The correlation coefficient was high for electrode sites nearby and decreased with increasing distance. For awake mice, the correlation was constantly lower than for mice under anesthesia. The variation within one electrode distance resulted from the different signals in the single recordings and from the various channel pairs. At electrode distance 0, the result of the correlation of the channels with themselves is given.

### Stable Long-Term Recordings of Electrophysiological and Spontaneous Astroglial Ca^2+^ Signals in the Mouse Cortex

**Figure 6:**
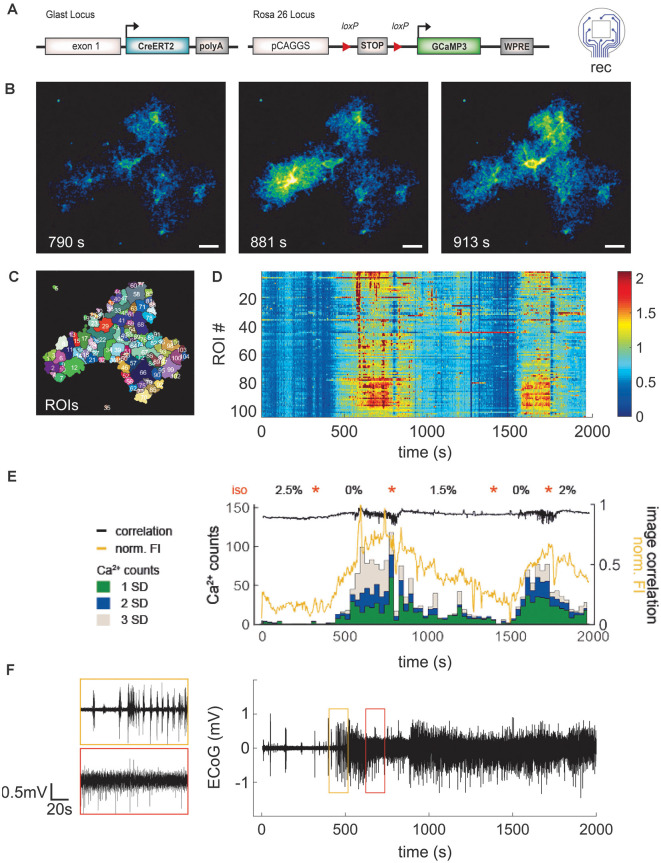
Application of LCP electrode for ECoG recording combined with 2P- Ca^2+^ imaging substantiates reduction of spontaneous astroglial Ca^2+^ signals under anesthesia. **(A)** Eight-channel ECoG recording and simultaneous 2P-Ca^2+^ imaging in transgenic mice with GCaMP3 expression in astrocytes. **(B)** 2P-LSM sequence displaying good cranial window quality with implanted LCP electrode 35 days post-surgery, scale bar = 20 μm. **(C)** Regions of interest (ROI) map computed by activity-dependent analysis using custom-made Matlab routines. **(D)** Heatmap illustrating normalized Ca^2+^ signal amplitudes [(*F* − *F*0)/*F*0] per ROI over time. Graphs **(D–F)** have the same time scale and isoflurane variations (orange stars). In contrast to the anesthetized state, awake mice display higher Ca^2+^ signal amplitudes. **(E)** Ca^2+^ events (counts) were grouped into small (1 SD), medium (2 SD), and large (3 SD) signals and merged with corresponding normalized fluorescence intensity of the image (FI, yellow trace) and image correlation (black trace). Anesthesia reduced particularly the frequency of medium and large Ca^2+^ signals which were highly abundant in awake animals. Increases of Ca^2+^events were accompanied by rises in fluorescence amplitude and variation of image correlation. Orange stars indicate transitions between isoflurane concentrations. **(F)** ECoG trace displayed as average over all channels shows clear transitions in brain activity depending on the anesthesia level.

To demonstrate the functionality of the combination of *in vivo* ECoG recording and Ca^2+^ events by 2P-LSM, an eight-channel electrode was implanted in a GLAST-Cre^ERT2^ × GCaMP3 mouse (Figure 6A). The depth of anesthesia was varied in an arbitrarily selected variation of isoflurane from 2.5% to 0% (orange stars in Figure 6E) to change the activity state, and thus the activity of neurons and astrocytes. We could achieve high-quality 2P-LSM and ECoG recordings over a long recording period (>30 min) and up to 35 days after electrode implantation. The cranial window quality allowed clear visibility of astroglial somata, processes, and Ca^2+^ transients (Figure 6B). The 2P-LSM images were processed with the MATLAB based tool MSparkles to find activity-based regions of interest (ROIs; Figure 6C), calculating the signal amplitudes [(*F* − *F*0)/*F*0; Figure 6D] and to classify the Ca^2+^ events in small (1 SD ≤ signal amplitude < 2 SD), medium (2 SD ≤ signal amplitude < 3 SD), and large signals (3 SD ≤ signal amplitude; Figure 6E). The standard deviation (SD) referred to the base fluorescence F0. The activity of the astrocytes increased with decreasing anesthesia which is in line with previous reports of other groups (Thrane et al., 2012; Bojarskaite et al., 2020).

To obtain a more comprehensive overview of the responses in a given field-of-view, the signal classification from the different ROIs was summed up within a time step of 30 s and the normalized FI, as well as the correlation of subsequent images, was calculated (Figure 6E). Under high anesthesia (2.5%) 0–0.2 events (Ca^2+^ counts) per second were detected, increasing up to 3.9 events (all classified signal types) per second in the awake mouse. This was paralleled by the normalized FI, which increased and decreased according to the inversely varying isoflurane concentration. The image correlation demonstrated fast Ca^2+^ signal changes (fast changing correlation result) during the awake state. Due to the image noise (which is different from image to image) the correlation coefficient is never exactly equal to 1. The average signal of the eight ECoG channels was calculated (Figure 6F). This signal allows for an exact determination of the actual depth of anesthesia during the 2P-imaging experiment or similar experimental procedures. Overall, the animal recovers rapidly from the anesthetized state but it lasts longer returning to the anesthetized condition after an awake episode.

## Discussion

Three different LCP surface micro-electrodes for electrical stimulation and ECoG recordings were developed and electrochemically optimized with platinum coating. Several application examples in anesthetized and awake mice demonstrated the versatility, convenience, and reliability of the novel LCP micro-electrodes to investigate brain function *in vivo*. Quality assurance (impedance measurements, microscopic control) was performed to sort out electrodes with production defects. In the animal studies, surface electrodes were easy to handle and showed excellent robustness. The investigations were not disrupted by electrode damages of any kind. The possibility of combined electrical stimulation or ECoG recording with 2P-imaging *in vivo* opens various opportunities to study neural function, e.g., to investigate neuron-glia interactions in the CNS.

### LCP Surface Electrodes Are Highly Biocompatible for Long-Term *In vivo* Observations

Biocompatibility of neural implants is a major concern in clinical application as well as research (Polikov et al., 2005). In contrast to the reported tissue response in rodents to intracortical electrodes (Minnikanti et al., 2010; Potter et al., 2012; Salatino et al., 2017), little information was obtained about the tissue response to epidural electrodes (Schendel et al., 2013; Shokoueinejad et al., 2019). Consequently, in this work, the macroscopic and cellular biocompatibility was tested under acute (3 days post-implantation) as well as chronic conditions (28 days post-implantation). Since the LCP electrodes were used in combination with cranial windows for 2P-imaging, we assessed the inflammatory response of glial cells by comparing animals with cranial window and electrode implant to simple cranial window surgery (sham). Neither astrocytes nor microglia displayed significant acute or chronic activation in LCP electrode carrying animals. In fact, sham animals showed an increased astroglial reactivity reflected by increased GFAP immunoreactivity, especially in the chronic application. Thus, the LCP surface electrode appears to display an even beneficial effect after cranial window surgery, likely due to the thickness of the electrodes (approx. 75 μm) filling the gap between the cortex and the glass coverslip. Thereby, the tissue may be stabilized and hemorrhages, as well as edema, may be reduced. In addition, no impact on the expression of the genetically encoded Ca^2+^ sensor GCaMP3 was detected, providing optimal conditions for 2P- Ca^2+^ imaging. An additional advantage of the LCP surface electrodes is the convenient use of their inner edges as a reference system for repetitive imaging of the same ROIs in chronic imaging experiments.

### Cortical Stimulation Activated Local and Global Network Activity

For *in vivo* stimulation of cortical tissue, the electrode arrays with the rectangular-shaped electrode sites were implanted in mice with GCaMP3 expression by principal neurons of the neocortex. The stimulation-evoked Ca^2+^ signals were dependent on stimulation amplitude and anesthesia level. With lower anesthesia, biphasic Ca^2+^ transients were observed, representing the consecutive activation of neuropil and somata. The direct effect of electrical stimulation mainly targets neuronal structures in cortical layer I with depolarizations further propagating to the somata in layer II/III. Simultaneously, the Ca^2+^ signal spreads intracellularly. The fluorescence intensity of the neuropil region (peak 1) did not show a significant relation to stimulation strength and anesthesia. The amplitude of the second peak involving the somata was mainly dependent on the stimulation intensity, and less on the anesthesia level. A stronger stimulation would elicit a stronger depolarization, leading to increased intracellular Ca^2+^ mobilization. A volatile anesthetic such as isoflurane acts on multiple targets. It reduces Na^+^ currents while inducing K^+^ currents, lowers resting membrane potential, depresses NMDA receptor-dependent excitatory transmission, and increases GABA_A_ receptor-mediated inhibitory transmission (Franks and Lieb, 1988; Rudolph and Antkowiak, 2004; Westphalen and Hemmings, 2006; Lissek et al., 2016; Zhao et al., 2019). Thus, neuronal excitability is considerably reduced, leading to the decreased opening probability of voltage-gated Ca^2+^ channels for example (Study, 1994; Koyanagi et al., 2019; Timic Stamenic et al., 2019). Hence, higher peak amplitudes are observed at lower isoflurane levels. The signal drop between activation of neuropil and soma was probably a local effect of activated Ca^2+^ pumps restoring the intracellular Ca^2+^ homeostasis, counteracting the active stimulation. In a recent study (Michelson et al., 2019), a Ca^2+^ signal decrease in neuronal soma and neuropil is described, supporting the finding of this study.

A signal undershoot after evoked Ca^2+^ transients has been previously reported (Majewska et al., 2000; Collins and Thomas, 2001). This phenomenon could reflect a state of depression after strong stimulation and depletion of intracellular Ca^2+^ stores. Reports of volatile anesthetics’ effect on ER and plasma membrane Ca^2+^ transport are controversial and less described in neurons than in myocytes for example (Blanck and Thompson, 1982; Collins and Thomas, 2001; Hannon and Cody, 2002). However, it is possible that the prolonged undershoot in anesthetized animals is caused by hyperactivity of the sarcoplasmic reticulum Ca^2+^ ATPase to refill the ER stores and delayed compensation by replenishing mechanisms. No significant changes were seen in the delay to the Ca^2+^ peaks nor the duration of the signal, suggesting a stereotypical neuronal Ca^2+^ response to stimulations (above the defined threshold). One could have expected an impact of anesthesia on the signal duration, given the fact that crucial Ca^2+^ extrusion mechanisms [Na^+^/Ca^2+^ exchanger (NCX) and the plasma membrane Ca^2+^ ATPase (PMCA)] are potentially inhibited by volatile anesthesia. Corresponding studies however were mainly performed *in vitro* or *ex vivo* (Franks et al., 1995; Ay et al., 2005).

### Astroglial Ca^2+^ Events Follow Neuronal Activation After Cortical Stimulation

To study astroglial Ca^2+^ responses after electrical stimulation, mice with astrocyte-specific GCaMP3 expression were used. The stimulation amplitude and anesthesia levels were varied. The rise of the Ca^2+^ transients started approximately 1.6 s to 2.6 s after stimulation onset and the maximum peak value was found between 3.5 s and 5.5 s, when the electrical stimulation was already terminated. Together with the delay time to the Ca^2+^ transient peaks of neurons (approximately 0.6 s to 1.2 s for neuropil and 2 s to 2.5 s for soma), the astroglial activation followed the neuronal activation, likely due to glial responses to the release of neurotransmitters (Araque, 2008; Volterra et al., 2014; Shigetomi et al., 2016; Guerra-Gomes et al., 2017). In addition, no dependency of the Ca^2+^ signal peak time to the anesthesia was found which was in line with the neuronal peak time. Similarly, astroglial Ca^2+^ peak amplitudes increased with augmenting stimulation intensity, as seen in neurons. However, this effect was less pronounced in astrocytes. Taken together with the observation of reduced delays to peak amplitude with increasing stimulation strength exclusively in astrocytes, the integrative function of astrocytes following neuronal activation is highlighted (Araque et al., 2014; Caudal et al., 2020). In accordance with neuronal Ca^2+^ signal duration, astroglial signal duration was not significantly modulated by stimulation intensity or isoflurane levels. In contrast to neurons, astrocytes displayed longer Ca^2+^ events with higher variability (3.6 ± 0.1 s vs. 8.4 ± 0.5 s; mean ± SD). Previous studies revealed prominent depression of astroglial Ca^2+^ signaling under anesthesia (Nimmerjahn et al., 2009; Thrane et al., 2012). In contrast to our experimental setup, these studies focused on spontaneous or physiologically evoked astroglial Ca^2+^ activity, thus accounting for the discrepancies.

### Multichannel Surface Electrodes Enabled the Recording of Electrical Signals in the Cortex

Determining the brain activity state of mice during complex *in vivo* experimental procedures can be crucial for successful and comprehensive data acquisition, e.g., by quantifying the depth of anesthesia (Land et al., 2012). With the LCP surface electrode arrays (round-shaped electrode sites), acquisition of low noise ECoG recordings was achieved *via* standard bandpass (0.5 Hz to 250 Hz) and notch filtering (50 Hz). No further signal filtering like wavelet denoising was required (Schweigmann et al., 2018). A channel similarity study was performed for the LCP electrodes by calculating the correlation of the individual channels. The result indicated that there was a high similarity between adjacent channels in the awake as well as in the anesthetized mouse. However, for more distant electrode sites, some signal differences were detected by decreasing channel correlation. Moreover, the channel correlation was systematically reduced in awake compared to anesthetized mice, reflecting the asynchronous network activity in awake states. In a recent study, channel correlations performed in mice and humans could resolve local activity patterns even when ECoG recording sites were less than 1 mm apart (Rogers et al., 2019).

Our results offer a valuable starting point for experimental designs involving evoked potentials, where ECoG activity and its propagation could be linked to functional 2P-LSM, thereby offering new options to study microcircuits across different cell populations (Mohajerani et al., 2013).

### Combination of *In vivo* 2P-LSM and ECoG Recordings to Unravel Neuron-Glia Interactions

The successful combination of *in vivo* 2P-LSM and ECoG recording was demonstrated in experiments with astroglial GCaMP3 expression. After 35 days post electrode implantation, the optical window visibility was of good quality, and low noise ECoGs could be acquired, enabling the identification of anesthesia depth. Analysis of Ca^2+^ transients showed a variation in the number of Ca^2+^ events per time when the anesthetics’ concentration was altered. Approximately 0.05–0.7 Ca^2+^ events/s in the anesthetized (isoflurane 1.5%) and 2.5–3.9 Ca^2+^ events/s in the awake mouse were detected. Previously a 10-fold reduction of spontaneous somatic signals was described for astrocytes when the mouse was anesthetized (Thrane et al., 2012). Considering the different field of views and physiological variations, we found comparable results. Thereby, the ECoG recordings will help to understand the temporal-spatial pattern of Ca^2+^-signals in future studies, which will link small ECoG changes with astroglial activity. Such experiments will be highly valuable to study neuron-glia interactions *in vivo*.

### Boundaries for Cortical Surface Electrode Application in Combination With 2P-LSM

Different technologies for the development of surface electrodes exist comprising various base materials like polyimide (Choi et al., 2010; Kuzum et al., 2014; Khodagholy et al., 2015; Vomero et al., 2020), parylene (Khodagholy et al., 2013; Park et al., 2014; Richner et al., 2014; Donahue et al., 2018) or less frequently LCP (Lee et al., 2009; Min et al., 2014). The use of LCP electrodes limited the visual access for 2P-LSM due to the non-transparent, white material, therefore one or several optical windows had to be integrated to enable successful access to the cortex. Another limitation of the selected technology was in the miniaturization of the electrode sites compared to other electrode technologies, where small electrode sites in the 20 μm range could be achieved (Kuzum et al., 2014; Khodagholy et al., 2015; Donahue et al., 2018). Further miniaturization might enhance the acquisition of asynchronous network activity. However, the industrial series production of the electrodes enabled a cost-effective solution which could become more and more important in decoding cell behavior and interaction *in vivo*.

Previously, thin, flexible, and transparent surface electrodes based on parylene C (with 16 channels and graphene-coated electrode sides and interconnections) were introduced for recording and stimulation application (Park et al., 2014, 2018). Fluorescent imaging had been performed at the cortical surface with one-photon excitation (UV light) over a cortex area of 4.6 × 3.4 mm^2^, so that the entire electrode was visible. With a transparency of more than 90% (Park et al., 2014), Ca^2+^ signals could be recorded directly at the electrode sites (Park et al., 2018). Also, a comparison of parylene C electrodes and platinum electrode sites was presented. The metal structure blocked the visual access, but the electrode impedance [|Z(*f* = 10 Hz)| ≈ 1 MΩ, 200 μm in diameter] was at least one-order smaller than the impedance of the graphene electrodes [|Z(*f* = 10 Hz)| ≈ 10 MΩ, 200 μm in diameter; Park et al., 2018]. Here, our circular platinum electrode sites showed an impedance range of |Z(*f* = 10 Hz)| = 29–48 kΩ for an electrode diameter of 150 μm. The CSC_C_ of the graphene electrodes was estimated to be approximately 88 μC/cm^2^ (Park et al., 2018), whereas we achieved a CSC_C_ of 10,500 μC/cm^2^ to 15,800 μC/cm^2^, indicating a higher current drive capability. A surface electrode made from PET, photoresist SU-8, gold electrode tracks, and graphene electrode sites was developed to use the transparent electrode for combined recording and deep tissue 2P-LSM (Thunemann et al., 2018). The impedance traces showed an impedance magnitude of |Z(*f* = 10 Hz)| ≈ 10 MΩ–200 MΩ for the size of the squared electrode sites of 100 μm × 100 μm (Thunemann et al., 2018). Again, the current drive capability of the graphene electrode site might be strongly limited.

Electrical recording and one photon or two-photon excitation in hippocampal tissue slices were combined using a polyimide electrode with graphene electrode sites (square-shaped 25 μm × 25 μm; Kuzum et al., 2014). The transparency of the electrode was characterized for light wavelength in the range of 400 nm to 900 nm and showed a limitation by the polyimide (thickness 12.5 μm and 25 μm) rather than graphene. For wavelengths above 600 nm, the transparency was 80% to 85% whereas between 600 nm and 450 nm it decreases down to 10% (Kuzum et al., 2014). This might be a major concern for deep tissue 2P-LSM where e.g., only weak signals (due to tissue scattering effects) can be acquired. Another recent study (Donahue et al., 2018) pointed out the result of the combination of an electrode array made from parylene C and gold interconnections with two-photon imaging. The square electrode sites had a size of 25 μm × 25 μm, and the width of the interconnection lines was around 20 μm at the electrode head (Donahue et al., 2018). The routing of the interconnection line was selected in a way that the visual access through the inner area of the electrode head was maximized. With that design, heating and photoelectric effects were minimized (Donahue et al., 2018). However, this could cause the problem that metal structures within the optical field might be heated up by laser light adsorption. This problem could also occur with polyimide electrodes.

Currently, there does not seem to be any electrode technology that can be used without restrictions. With the high-level microsystem-produced electrodes, a high degree of miniaturization and improved flexibility would be possible. However, general transparency could be only achieved if the electrode contacts were also realized with transparent conductive materials. But these materials still seem to have deficits in electrode impedance and current transfer capability.

## Conclusion

We developed versatile, reliable, and cost-effective LCP surface electrodes, allowing the combination of *in vivo* 2P-imaging and electrophysiology in the mouse CNS. The pilot studies highlighted the biocompatibility and the new opportunities provided by the technology. Several types of LCP electrodes, optimized for tissue stimulation and low noise ECoG recording, are available to study and modulate CNS function in health and disease.

## Data Availability Statement

The original contributions presented in the study are included in the article, further inquiries can be directed to the corresponding author.

## Ethics Statement

The animal study was reviewed and approved by Landesamt für Gesundheit und Verbraucherschutz of Saarland state (license numbers: 71/2013, 36/2016).

## Author Contributions

MS developed and optimized surface electrodes, performed imaging and recording, data analysis, wrote the initial manuscript, and conceived the study. LC performed the immunohistochemical assessment of biocompatibility, electrode implantation surgeries (optimization and preparation), imaging and recording, data analysis, wrote initial manuscript, and conceived the study. GS provided custom-made Matlab routines (MSparkles). AS and KK conceived the study and revised the manuscript. FK conceived the study, provided infrastructure, grant support, and finalized the manuscript. All authors contributed to the article and approved the submitted version.

## Conflict of Interest

The authors declare that the research was conducted in the absence of any commercial or financial relationships that could be construed as a potential conflict of interest.

## Publisher’s Note

All claims expressed in this article are solely those of the authors and do not necessarily represent those of their affiliated organizations, or those of the publisher, the editors and the reviewers. Any product that may be evaluated in this article, or claim that may be made by its manufacturer, is not guaranteed or endorsed by the publisher.

## Abbreviations

2P-LSM: two-photon laser scanning microscopy
CNS: central nervous system
Cre: Cre DNA recombinase
CreERT2: Cre DNA recombinase fused to mutated human estrogen receptor ligand-binding domain
CSCc: cathodic charge storage capacity
DAPI: 4′,6-diamidino-2-phenylindole
ECoG: electrocorticogram
FI: fluorescence intensity
GFAP: glial fibrillary acidic protein
GFP: green fluorescent protein
GLAST: glutamate/aspartate transporter
Iba1: ionized calcium binding adaptor molecule 1
LCP: liquid crystal polymer
loxP: locus of crossover of the bacteriophage P1
NCX: Na^+^/Ca^2+^ exchanger
Nex: NeuroD6, subfamily of neuronal basic helix-loop-helix transcription factors
NMDA receptor: *N*-methyl-d-aspartate receptor
pCAGGS: cytomegalovirus immediate early enhancer/chicken beta-actin/rabbit beta-globin hybrid promoter
PMCA: plasma membrane Ca^2+^ ATPase
ROI: region of interest
SD: standard deviation
SERCA: sarcoplasmic/endoplasmic reticulum Ca^2+^ ATPase
Th: Threshold
WPRE: Woodchuck hepatitis virus posttranscriptional regulatory element
Z: impedance.
